# Distribution of Pathogens and Predictive Values of Biomarkers of Inflammatory Response at ICU Admission on Outcomes of Critically Ill COVID-19 Patients with Bacterial Superinfections—Observations from National COVID-19 Hospital in Croatia

**DOI:** 10.3390/diagnostics12092069

**Published:** 2022-08-26

**Authors:** Maja Ćurčić, Marko Tarle, Hani Almahariq, Sonja Hleb, Juraj Havaš, Marko Pražetina, Hrvoje Lasić, Emil Dolenc, Andrea Kukoč, Antonija Mihelčić, Ivan Miko, Andrea Romić, Danijela Tipura, Željka Drmić, Marcela Čučković, Vanja Blagaj, Ivica Lukšić, Jasminka Peršec, Andrej Šribar

**Affiliations:** 1 Clinical Department for Anesthesiology, Reanimatology and Intensive Care Medicine, University Hospital Dubrava, 10000 Zagreb, Croatia; 2 School of Dental Medicine, University of Zagreb, 10000 Zagreb, Croatia; 3 Department of Maxillofacial Surgery, University Hospital Dubrava, 10000 Zagreb, Croatia; 4 School of Medicine, University of Zagreb, 10000 Zagreb, Croatia

**Keywords:** COVID-19, intensive care medicine, superinfection, survival analysis

## Abstract

Background: Superinfections contribute to mortality and length of stay in critically ill COVID-19 patients. The aim of this study was to determine the incidence and pathogen distribution of bacterial and fungal superinfections of the lower respiratory tract (LRTI), urinary tract (UTI) and bloodstream (BSI) and to determine the predictive value of biomarkers of inflammatory response on their ICU survival rates. Methods: A retrospective observational study that included critically ill COVID-19 patients treated during an 11-month period in a Croatian national COVID-19 hospital was performed. Clinical and diagnostic data were analyzed according to the origin of superinfection, and multivariate regression analysis was performed to determine the predictive values of biomarkers of inflammation on their survival rates. Results: 55.3% critically ill COVID-19 patients developed bacterial or fungal superinfections, and LRTI were most common, followed by BSI and UTI. Multidrug-resistant pathogens were the most common causes of LRTI and BSI, while *Enterococcus faecalis* was the most common pathogen causing UTI. Serum ferritin and neutrophil count were associated with decreased chances of survival in patients with LRTI, and patients with multidrug-resistant isolates had significantly higher mortality rates, coupled with longer ICU stays. Conclusion: The incidence of superinfections in critically ill COVID-19 patients was 55.3%, and multidrug-resistant pathogens were dominant. Elevated ferritin levels and neutrophilia at ICU admission were associated with increased ICU mortality in patients with positive LRTI.

## 1. Introduction

The COVID-19 pandemic, which originated in Wuhan, China in late 2019, and in early 2020 spread throughout the planet due to the high transmission potential of SARS-CoV-2 [1], affected healthcare systems in almost every country so severely that it was declared a public health emergency by the World Health Organization (WHO) [2,3]. In Croatia, 300,000 cases were recorded in 2020 [4], out of which approximately 10% needed to be hospitalized, while others presented with mild symptoms similar to influenza. Of those patients that are hospitalized, approximately 10% require intensive care unit (ICU) admission due to a severe course of disease caused by dysregulated immune response [5] leading to coagulopathy [6], massive alveolar damage [7], and progressive respiratory failure, which is often so severe that mechanical ventilation is necessary in order to achieve adequate gas exchange [8,9], all of which are linked to adverse outcomes [10].

Superinfections in critically ill COVID-19 patients are common due to various factors, such as the fact that SARS-CoV-2 may enhance colonization and attachment of bacteria to host tissue, not only during the illness itself, but also in the post-COVID phase [11]. Airway dysfunction, cytopathology and tissue destruction induced by SARS-CoV-2 infection or during bacterial co-infection may facilitate systemic dissemination of the virus and/or bacterial co-pathogens, dramatically increasing the risk of blood infections and sepsis [12,13]. In addition, due to the widely accepted therapeutic regimen of corticosteroid therapy, which reduces COVID-19 mortality [14] but may promote immunosuppressive states, [15,16] combined with irrational use of antibiotics (in hospital but unfortunately also before hospital admission) [17], certain ICU stay (non-COVID-19) factors should be also taken into account as contributing factors leading to increased incidence of superinfections: presence of intravascular [18] and urinary [19] catheters. Multidrug-resistant pathogen infections are of special concern in these patients due to their deleterious effect on ICU and hospital stays and mortality [16,20].

The goal of this study was to determine the incidence and pathogen distribution of bacterial and fungal superinfections of the lower respiratory tract, urinary tract and bloodstream and to determine the predictive value of biomarkers of inflammatory response on ICU survival rates for each origin of infection in critically ill COVID-19 patients treated in a national COVID-19 hospital.

## 2. Materials and Methods

This study was designed as a retrospective observational study, and it included patients admitted to the combined intensive care unit (ICU) organized in national COVID-19 hospital Dubrava UH [21] through an 11-month period, during which the hospital was repurposed to function as a COVID-19-only hospital.

After institutional ethics board approval (ID: 2021/2309-01), data collection from the hospital information system (iBIS, IN2, Zagreb, Croatia) was performed. Recorded variables were: basic demographic characteristics (gender, age), relevant comorbidities from which Charlson comorbidity index (CCI) was calculated; laboratory parameters at ICU admission, including white blood cell count (WBC, ×10^9^/L), neutrophil and lymphocyte percentage in WBC, as well as neutrophil/lymphocyte ratio (NLR), serum ferritin (µg/L), serum procalcitonin (PCT, ng/mL), serum C-reactive protein (CRP, mg/L), and serum interleukin 6 (IL-6, pg/mL); sequential organ failure assessment (SOFA) score, and duration of ICU stay and mortality rates.

In terms of microbiology sample acquisition and analysis, LRT pathogens were isolated from bronchoscopy-guided aspiration after lavage with 20 mL of sterile 0.9% NaCl. Blood cultures were considered positive if growth was recorded from two independent sampling sites (vascular catheter plus direct venipuncture after sterile skin preparation), and urine cultures were sampled by direct aspiration through the urinary catheter sampling port after sterile preparation, per institutional microbiology department specimen sampling protocol.

### Statistical Analysis

Data are presented as tables and charts. Continuous variables are displayed as either mean and standard deviation (SD) for values with Gaussian distribution, or median and interquartile range or 95% confidence interval (CI) for data that do not follow normal distribution. Normality of distribution was assessed using the Shapiro–Wilk test. Categorical variables are displayed as counts and percentages.

Differences in independent continuous variables were tested for statistical significance using Student’s *t* test for independent samples or Mann–Whitney U test, depending on the distribution of data. For dependent (repeated measurement on day 7) variables, paired sample *t* test or Wilcoxon rank test were used to test for statistical significance.

For more than two groups, two-way analysis of variance (ANOVA) was used to test for significance when comparing normally distributed groups and Kruskal–Wallis test was used for variables without normal distribution in order to avoid variability in type I error rate. The Dwass-Steel-Critchlow-Fligner method was used to perform pairwise comparisons between groups.

Differences in categorical variables were tested for statistical significance using χ^2^ or Fisher’s exact test for 2 × 2 tables.

Multivariable logistic regression, adjusted for age [22], sex [23] and Charlson comorbidity index [24], as well for interactions between biomarkers, was performed in order to determine the effect of inflammatory parameters on ICU admission and ICU mortality rates for patients with bacterial pneumonia and positive blood cultures. Fit of the model was evaluated using the Hosmer–Lemeshow goodness of fit test and Nagelkerke R2 statistic and model ROC AUC value. The model was tested for multicollinearity, and the neutrophil/lymphocyte ratio (NLR) was excluded from the model due to a variance inflation factor (VIF) > 5 [25,26]. For patients with urinary infections, the regression model was rejected due to high VIF (>10) for over 50% of the variables and low model fit (R^2^ < 0.1).

Survival times were plotted using the Kaplan–Meier method, and survival probability between groups was tested using the Mantel–Cox log-rank test with pairwise comparisons and expressed as hazard ratio (HR) and 95% CI.

*p* values < 0.05 were considered statistically significant. Software packages used for statistical analysis and data visualization were jamovi v2.3 [27] with survminer 0.3.0 [28] and finalfit 1.0 [29] modules and JASP v0.14.1 [30].

## 3. Results

During an 11-month window, 692 patients were admitted to Dubrava UH COVID-19 ICUs, and 383 (55.3%) developed bacterial superinfections with positive bacterial or fungal isolates.

Of those patients, 79.6% developed growth in lower respiratory tract (LRT) cultures, 34.8% had positive blood cultures, 31.2% had positive urine cultures, and 11% had growth in other samples (pleural fluid, cerebrospinal fluid, or skin and soft tissue swabs).

In the studied cohort with bacterial superinfections, 66.3% were males, and the most important comorbidities present at admission were arterial hypertension (71%), diabetes mellitus (34.3%), congestive heart failure (14.9%), and renal failure (14.9%, of which 7.5% were end-stage patients receiving renal replacement therapy).

In patients with bacterial superinfections, time from first positive SARS-CoV-2 PCR test to ICU admission was longer (median 5, IQR 2–10 days) than for those without (median 4, IQR 1–8 days).

Distribution of cultures according to origin of infection and identified pathogens for each site of origin are listed in Table 1.

### 3.1. Patients with Lower Respiratory Tract Infections

In terms of hospital and/or ventilator acquired bacterial pneumonia, out of 305 patients with positive LRT growth, 295 (96.7%) were mechanically ventilated, while 262 mechanically ventilated patients from the cohort (47.1% of all patients receiving invasive mechanical ventilation) did not acquire HAP (*p* < 0.001).

Ten (3.3%) patients had antibiotic-susceptible Gram-positive isolates, 18 (5.9%) had antibiotic-susceptible Gram-negative isolates, three (1%) had combined antibiotic-susceptible isolates, 267 (87.5%) had MDR isolates and seven (2.3%) had fungal pneumonia.

There was no significant difference in age between patients that acquired VAP/HAP compared to those that did not (71, IQR 63–79 vs. 72, IQR 64–78 years, *p* = 0.729), nor in incidence of HAP/VAP between females and males (39% females vs. 46.6% males, *p* = 0.061). There was also no statistically significant difference in CCI between patients that developed VAP/HAP compared to those that did not (4, IQR 3–6 vs. 5, IQR 3–7, *p* = 0.062), nor between subgroups (*p* = 0.131).

Patients with HAP/VAP had significantly longer durations between first positive RT-PCR test and ICU admission (5.5, IQR 2–10 vs. 4, IQR 1–8 days, *p* > 0.001) and were more often admitted from hospital wards or external ICUs (51.6% of all patients were admitted from wards, 44.8% from external ICUs, and 33.6% from emergency department, *p* < 0.001). Duration of ICU stay was also significantly longer in patients with HAP/VAP compared to those without growth, (13, IQR 9–18 vs. 6 IQR 3–10 days, *p* < 0.001).

Differences in serum inflammatory marker levels at admission, according to type of causative microorganism in patients with HAP or VAP, are displayed in Table 2. While there were statistically significant differences between groups in terms of IL-6 levels, after post hoc DSCF correction was performed, significance was lost. At day 7, there were no statistically significant differences in serum inflammatory marker levels found between groups or timepoints.

After multivariable adjustment, only neutrophil percentage in WBC count (OR 1.10, IQR 1.03–1.29 per 1% increase) and ferritin serum level (OR 1.13, IQR 1.03–1.25 per each 10 mcg/L increase) at ICU admission had statistically significant predictive value for ICU mortality (Table 3.).

SOFA score at admission was 3.5 (3.2–3.9, 95% CI) for patients without bacterial pneumonia, 2.4 (0.7–4.1, 95% CI) for patients with Gram-positive antibiotic-susceptible pathogens, 4.8 (3.5–6.1, 95% CI) for patients with Gram-negative antibiotic-susceptible pathogens, 6.5 (3.3–9.6, 95% CI) for patients with combined antibiotic-susceptible pathogens, 3.9 (3.7–4.3, 95% CI) for patients with multidrug-resistant pathogens and 4 (2.3–5.7, 95% CI) for patients with fungal pneumonia, without statistically significant differences between groups. The only statistically significant difference at day 7 compared to admission was found for patients without pneumonia (4, IQR 2–6) and those with MDR pneumonia (5, IQR 3–7). No differences were found between groups at day 7.

Associations between ICU mortality rates and type of isolated pathogen are displayed in Table 4. Patients without VAP/HAP and those with antibiotic-susceptible Gram-negative isolates had significantly lower mortality compared to other groups. Median survival for patients without LRT growth was 9 (8–9, 95% CI) days, 12 (3–N/A, 95% CI) days for patients with Gram-positive antibiotic-susceptible pathogens, 9 (7–N/A, 95% CI) days for patients with Gram-negative antibiotic-susceptible pathogens, 10 (1–N/A, 95% CI) days for patients with combined antibiotic-susceptible pathogens, 14 (13–15, 95% CI) days for patients with multidrug-resistant pathogens, and 15 (7–N/A, 95% CI) days for patients with fungal pneumonia.

**Figure 1:**
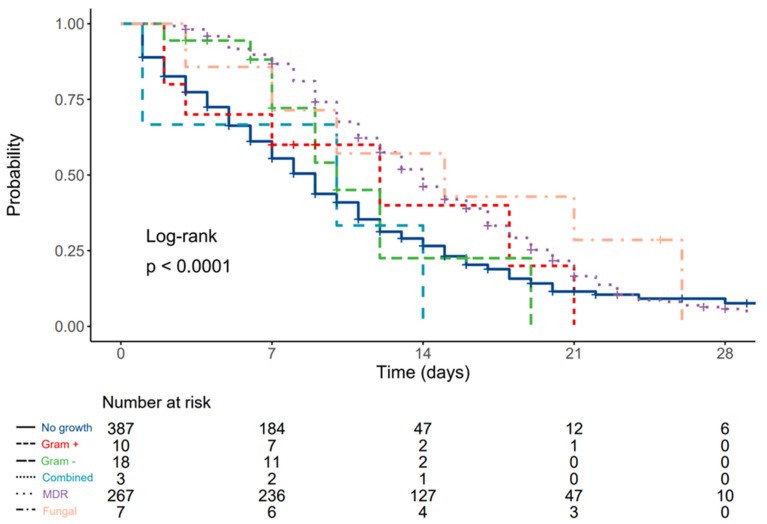
Kaplan–Meier plot depicting differences in survival times according to type of LRT pathogen.

Compared to patients without LRT growth, the hazard ratio (HR) was 0.84 (0.40–1.79, *p* = 0.658) for patients with Gram-positive antibiotic-susceptible pathogens, 0.88 (0.47–1.65, *p* = 0.689) for patients with Gram-negative antibiotic-susceptible pathogens, 1.40 (0.45–4.37, *p* = 0.565) for patients with combined antibiotic-susceptible pathogens, 0.58 (0.48–0.69, *p* < 0.001) for patients with multidrug-resistant pathogens, and 0.46 (0.20–1.04, *p* = 0.061) for patients with fungal pneumonia. (Figure 1).

### 3.2. Patients with Bloodstream Infections

Out of 133 patients with isolated blood culture pathogens, 26 had antibiotic-susceptible Gram-positive isolates, two had antibiotic-susceptible Gram-negative isolates, four had combined antibiotic-susceptible isolates, 100 had MDR isolates, and one tested positive for fungi in blood culture.

There was no significant age difference between patients that acquired BSI compared to those that did not (72, IQR 64–79 vs. 71, IQR 64–79 years, *p* = 0.125), nor difference in the incidence of BSI between females and males (18% females vs. 19.8% males, *p* = 0.563).

There was no statistically significant difference in CCI between patients that developed BSI compared to those who did not (*p* = 0.090), but patients with MDR isolates had higher CCI scores compared to those with susceptible Gram-negative isolates (4, IQR 3–6 vs. 4 IQR 2–2, *p* = 0.001).

There was no significant difference in duration between first positive RT-PCR test and ICU admission (5, IQR 2–10 vs. 5, IQR 1–9 days, *p* = 0.07) for patients with BSI, but they were more often admitted from hospital wards or external ICUs (22.2% of all of the patients were admitted from wards, 24.6% from external ICUs, and 12.2% from emergency department, *p* = 0.003).

Duration of ICU stay was also significantly longer in patients with BSI compared to those without growth (14, IQR 9–20 vs. 8 IQR 4–12 days, *p* < 0.001).

Differences in serum inflammatory marker levels according to type of causative microorganism in patients with positive blood cultures are displayed in Table 5. While there were statistically significant differences between groups in terms of serum procalcitonin levels, after post hoc DSCF correction was performed, significance was lost.

On day 7, compared to admission, a statistically significant drop was found in serum lymphocyte levels for patients without BSI (*p* = 0.032) and those with MDR BSI (*p* = 0.026), as well as an increase in NLR in patients without BSI (*p* = 0.004). Additionally, a significant drop in CRP was observed in patients without BSI (*p* < 0.001), and significantly higher IL-6 levels were measured in patients with Gram-negative infections compared to other groups (*p* < 0.05 vs. other groups).

SOFA score at admission was 3.9 (3.6–4.1, 95% CI) for patients without bloodstream infections, 3.2 (2.2–4.2, 95% CI) for patients with Gram-positive antibiotic-susceptible blood cultures, 6.5 (3.3–9.7, 95% CI) for patients with Gram-negative antibiotic-susceptible pathogens, 2.3 (0–4.9, 95% CI) for patients with combined antibiotic-susceptible pathogens, 3.6 (3.2–4.1, 95% CI) for patients with MDR isolates, and 4 (0–8.5, 95% CI) for patients with fungal BSI, without statistically significant differences between groups.

Compared to admission, a statistically significant SOFA difference at day 7 was found for patients without bloodstream infections (4.9, 4.6–5.3 95% CI, *p* < 0.001) and for patients with MDR isolates (5.3, 4.7–5.9 95% CI, *p* < 0.001), and at day 7, patients with Gram-negative susceptible isolates had significantly higher SOFA scores compared to all groups except for that with fungal BSI.

Associations between ICU mortality and bloodstream pathogens are displayed in Table 6. No statistically significant difference between groups was found. Additionally, no significant effect of any of the measured biomarkers at ICU admission on ICU mortality was found in the multivariate regression model (Table 7).

Median survival for patients without bloodstream infections was 10 (9–11, 95% CI) days, 15 (7–N/A, 95% CI) days for patients with cultivated Gram-positive antibiotic-susceptible pathogens, 9.5 (7–N/A, 95% CI) days for patients with cultivated Gram negative antibiotic-susceptible pathogens, 15 (5–N/A, 95% CI) days for patients with combined antibiotic-susceptible pathogens, 15 (14–18, 95% CI) days for patients with multidrug-resistant bacterial pathogens, and 12 (N/A–N/A, 95% CI) days for patients with fungal isolates.

**Figure 2:**
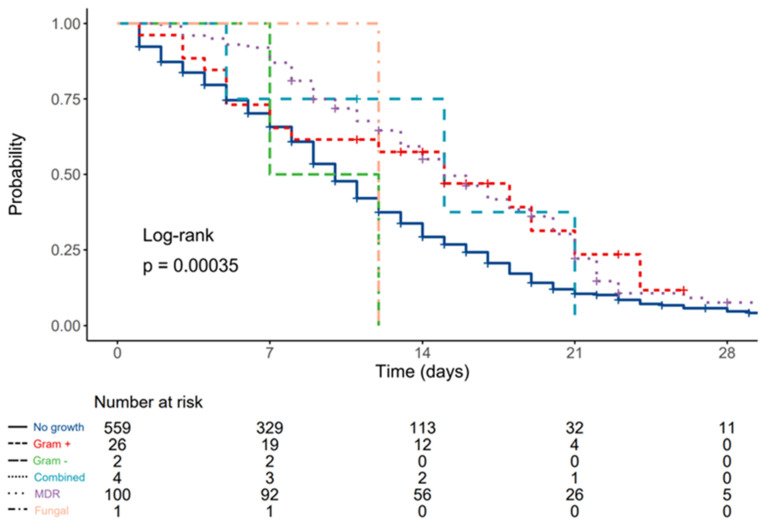
Kaplan–Meier plot depicting differences in survival times according to type of blood culture pathogen.

Compared to patients without bloodstream infections, HR was 0.59 (0.36–0.96, *p* = 0.034) for patients with Gram-positive cultures, 1.59 (0.40–6.39, *p* = 0.513) for patients with Gram-negative cultures, 0.66 (0.21–2.05, *p* = 0.470) for patients with combined pathogens, 0.60 (0.47–0.76, *p* < 0.001) for patients with MDR isolates, and 1.13 (0.16–8.03, *p* = 0.905) for patients with fungal blood isolates (Figure 2).

### 3.3. Patients with Positive Urine Cultures

Out of 120 patients with positive urine cultures, 21 had antibiotic-susceptible Gram-positive isolates, 22 had antibiotic-susceptible Gram-negative isolates, four had combined antibiotic-susceptible isolates, 51 had MDR isolates, and 22 tested positive for fungus in blood culture.

There were significantly more females with urinary infections compared to males (25% vs. 13.6%, *p* < 0.001), without significant age difference (72, IQR 66–79 vs. 72, IQR 63–68, *p* = 0.667).

No significant difference in CCI scores was found between patients who developed urinary infections compared to those who did not (*p* = 0.847), nor between patients with different subgroups of urinary pathogens (*p* = 0.701).

Patients with bacterial growth in urine cultures had statistically significant longer duration between first positive RT-PCR test and ICU admission (5, IQR 2–11 vs. 5, IQR 1–9 days, *p* = 0.027), and were more often admitted from hospital wards or external ICUs (18.4% of all of the patients were admitted from wards, 24.6% from external ICUs, and 11.8% from emergency department, *p* = 0.006).

Duration of ICU stay was also significantly longer in patients with bacterial/fungal growth in urine cultures compared to those without growth (12.5, IQR 9–18 vs. 8, IQR 4–13 days, *p* < 0.001).

Differences in serum inflammatory markers between groups are displayed in Table 8. No significant differences were found between groups.

On day 7, patients without isolated pathogens had significant WBC and NLR increases, as well as lymphocyte count drop (*p* < 0.001), and patients with MDR urine isolates had significantly higher neutrophil counts compared to those without growth (*p* = 0.049).

SOFA score at admission was 3.8 (3.5–4.0, 95% CI) for patients without urinary infections, 4.1 (3.0–5.3, 95% CI) for patients with Gram-positive antibiotic-susceptible isolates, 2.9 (1.8–4.1, 95% CI) for patients with Gram-negative antibiotic-susceptible isolates, 2.0 (0–4.6, 95% CI) for patients with combined antibiotic-susceptible pathogens, 3.8 (3.1–4.4, 95% CI) for patients with MDR isolates, and 5.5 (4.5–6.4, 95% CI) for patients with fungal growth in urinary samples, with significantly higher scores in patients with fungal growth compared to those with Gram-negative and sterile isolates.

Associations between ICU mortality and urinary pathogens are displayed in Table 9. No statistically significant difference between groups was found.

Median survival was 10 (9–11, 95% CI) days for patients without urinary infections, 17 (12–NA, 95% CI) days for patients with Gram-positive antibiotic-susceptible isolates, 13 (12–NA, 95% CI) days for patients with Gram-negative antibiotic-susceptible isolates, 20 (5–NA, 95% CI) days for patients with combined antibiotic-susceptible pathogens, 14 (12–18, 95% CI) days for patients with MDR isolates, and 13 (10–21, 95% CI) days for patients with fungal growth in urinary samples.

**Figure 3:**
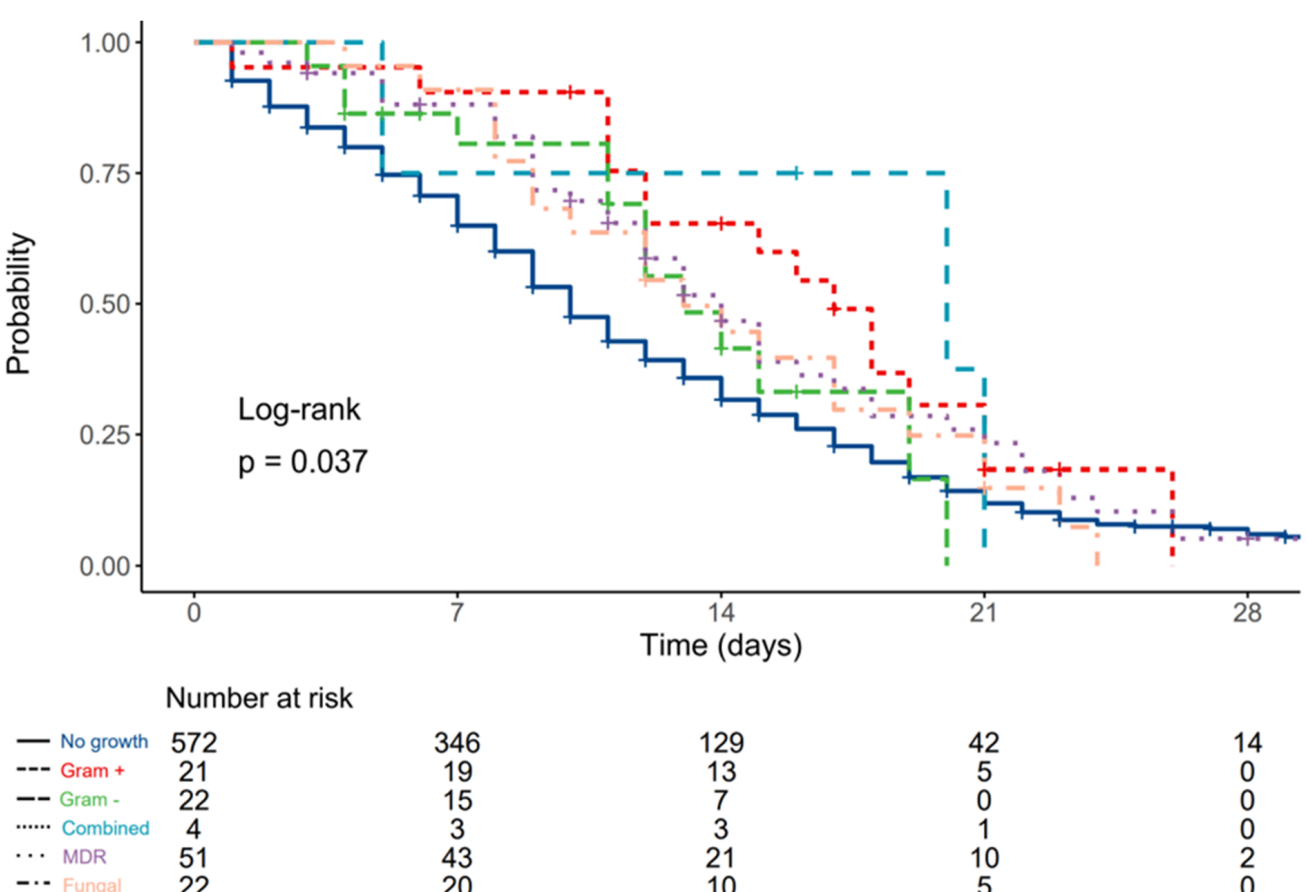
Kaplan–Meier plot depicting differences in survival times according to type of urinary culture pathogen.

Compared to patients without urinary infections, HR was 0.56 (0.34–0.93, *p* = 0.025) for patients with Gram-positive antibiotic-susceptible isolates, 0.75 (0.43–1.30, *p* = 0.307) for patients with Gram-negative antibiotic-susceptible isolates, 0.54 (0.17–1.68, *p* = 0.285) for patients with combined antibiotic-susceptible isolates, 0.71 (0.52–0.98, *p* = 0.036) for patients with MDR isolates, and 0.78 (0.50–1.23, *p* = 0.284) for patients with fungal growth in urinary samples (Figure 3).

## 4. Discussion

The results of this observational study show that multidrug-resistant pathogens (with *Acinetobacter baumanii* singled out as the most common one, and MRSA second most common) were the most common causes of bacterial superinfections of the lower respiratory tract and bloodstream, and *Enterococcus faecalis* the most common pathogen causing urinary superinfections in critically ill COVID-19 patients treated in UH Dubrava. Serum ferritin and neutrophil count were associated with decreased chance of survival in patients with positive LRT isolates, and patients with multidrug-resistant isolates had significantly higher mortality rates compared to other subgroups.

In a previously published analysis by Kukoč et al., which included all of the patients (both with and without superinfections) treated in our center, the SOFA score, PaO_2_/FiO_2_ at ICU admission, and history of arterial hypertension had an effect on ICU mortality, as well as the need to initiate invasive mechanical ventilation after multivariate adjustment. In survivors, an increase in PaO_2_/FiO_2_ after the first 7 days was observed, while a decrease applied to SOFA [10].

The percentage of patients with *Acinetobacter baumanii* superinfections in our cohort was significantly higher compared to the results of a review by Pasero et al. [16], where it varied between 0.8–61%, with most studies reporting results between 30 and 40%. The most probable reason for the reported prevalence of *Acinetobacter baumanii* is the fact that compared to other centers where ICU beds were located in ICUs, in UH Dubrava there was an increased number of ICU beds (85 beds in total, of which around one third were located in rooms that were repurposed to become makeshift ICUs) and reduced nurse/patient ratio, which would need to be 1:1 in mechanically ventilated patients but was 1:2.5–1:3 [31,32].

There were variations in the percentage of patients with MDR infections during the 11 month observation window, which were more apparent when a higher percentage of beds were occupied, once again confirming the importance of maintaining a lower nurse/patient ratio as a part of strategy to reduce the incidence of MDR infections in the ICU.

Since in the same hospital, with the same prevention bundles in place, the incidence of MDR infections was significantly lower before the COVID-19 pandemic [33], these factors, together with immune system dysregulation [5], may provide a probable explanation for the high *Acinetobacter baumanii* prevalence in these patients.

While there was no predictive value of any measured biomarker at ICU admission on outcomes in the multivariable model in patients with bloodstream infections, a significant association was found between ICU mortality and neutrophil percentage in WBC and serum ferritin levels, respectively, in patients with LRT superinfections (HAP/VAP). For each 10 mcg/mL increase in serum ferritin, there was a 13% higher probability, and for each % increase in neutrophil ratio in WBC, there was a 10% higher probability of death in the ICU. The observed effects of ferritin levels on mortality were in concordance with results published by Abers at al. [34], but it must be noted that the aforementioned article was not limited to ICU patients and included a wider spectrum of biomarkers.

The role of neutrophils in the COVID-19 pathophysiological mechanism is well observed, with the generation of neutrophil extracellular traps (NETs, which promote lung epithelial cell death in vitro [35]), the presence of various subsets of neutrophils (immature, immunosuppressive and activated) in the circulation, and lung infiltrates [36]. Compared to meta-analyses by Henry et al., in which leukocytosis, neutrophilia, lymphopenia, thrombocytopenia and elevated IL-6, IL-10 and ferritin levels were associated with worse outcomes [37,38], our results suggest that only elevated ferritin levels and neutrophilia were associated with increased ICU mortality. It should be noted that only patients with severe clinical presentation treated in the ICU were included in our analysis.

While many studies investigated the effect of neutrophil to lymphocyte ratio on COVID mortality rates, which was well established in a meta-analysis by Wang et al. [39], it was not included in the final regression model due to multicollinearity (high VIF), but there was no significant association with mortality in univariate regression analysis.

Compared to the survival analysis by He et al. [40], in our study, hazard ratios were generally lower for patients with bacterial superinfections, but with higher mortality rates. This might be explained by the fact that our study included only critically ill patients of much higher age, with multiple comorbidities and lower survival rates, as described by Kukoč et al. [10].

Finally, while the results of observational studies such as this one and many similar that included various subpopulations of COVID-19 patients do provide insight on clinical characteristics and predictors of outcome, their utility in diagnostic and treatment decisions will be fully understood after they are analyzed with multicriteria decision aid tools such as PROMETHEE [41] and ELECTRA-MOr [42,43].

There were certain limitations of this study. First and foremost, the number of analyzed biomarkers was not as wide in scope as in some other trials (such as that by Abers et al.).

One other possible limitation was the fact that therapeutic regimen (exact doses of corticosteroids, anticoagulation and anti-aggregation therapy, antiviral, and immunomodulatory drugs) was not recorded electronically but rather on paper charts that were sealed after patient discharge due to COVID containment measures and could not be included in the analysis. However, it should be noted that the COVID-19 related therapeutic regimen was uniform in all patients because of Croatian ministry of health COVID-19 treatment guidelines, with only minor deviations from protocol (such as enoxaparin vs. dalteparin as LMWH of choice, or dexamethasone vs. methylprednisolone as steroid of choice).

## 5. Conclusions

The incidence of superinfections in the observed cohort of critically ill COVID-19 patients was 55.3% with multidrug-resistant pathogen strains (mostly *A. baumannii*) dominant in LRTI and BSI, and *Enterococcus faecalis* was the most common UTI pathogen. In a multivariate regression analysis, elevated ferritin levels and neutrophilia at ICU admission were associated with increased ICU mortality in patients with positive LRTI, but no association was found in patients with BSI.

## Figures and Tables

**Table 1:** Distribution of pathogens according to origin of infection.

Microorganism	LRT	LRT ^2nd^	BSI	BSI ^2nd^	Urine	Other
*Streptococcus pneumoniae*	2 (0.7 %)	Ø	Ø	Ø	Ø	Ø
*MSSA*	8 (2.7 %)	9 (5.5%)	5 (4.3%)	7 (16.3%)	Ø	Ø
*MRSA*	31 (10.6 %)	49 (30.1%)	12 (10.3%)		1 (0.9%)	5 (13.9%)
*E. Coli*	3 (1.0 %)	3 (1.8%)	3 (2.6%)	1 (2.3%)	16 (13.9%)	
*Klebsiella pneumoniae*	14 (4.8 %)	25 (15.3%)	8 (6.9%)	6 (14%)	10 (8.7%)	3 (8.3%)
*Klebsiella pneumoniae ESBL*	2 (0.7 %)	1 (0.6%)	Ø	1 (2.3%)	1 (0.9%)	Ø
*Klebsiella pneumoniae OXA-48*	5 (1.7 %)	6 (3.7%)	Ø	Ø	Ø	1 (2.8%)
*Enterococcus faecalis*	Ø	4 (2.5%)	6 (5.2%)	8 (18.6%)	24 (20.9%)	4 (11.1%)
*Enterobacter cloacae*	1 (0.3 %)	3 (1.8%)	Ø	1 (2.3%)	1 (0.9%)	1 (2.8%)
*Acinetobacter baumannii*	195 (66.8 %)	27 (16.6%)	76 (65.5%)	4 (9.3%)	23 (20%)	11 (30.6%)
*VRE*	Ø	Ø	Ø	Ø	2 (1.7%)	4 (11.1%)
*Citrobacter freundii*	1 (0.3 %)	1 (0.6%)	Ø	Ø	Ø	Ø
*Achromobacter xylosoxidans*	1 (0.3 %)	Ø	Ø	Ø	Ø	Ø
*Pseudomonas aeruginosa*	19 (6.5 %)	26 (16.6%)	1 (0.9%)	6 (14%)	3 (2.6%)	3 (8.3%)
*Proteus mirabilis*	2 (0.7 %)	3 (1.8%)	Ø	Ø	4 (3.5%)	2 (5.6%)
*Candida albicans*	7 (2.4 %)	4 (2.5%)	Ø	Ø	17 (4.8%)	Ø
*Candida non-albicans*	Ø	Ø	1 (0.9%)	Ø	3 (2.6%)	Ø
*Morganella* spp.	1 (0.3 %)	2 (1.2%)	Ø	Ø	Ø	Ø
*Enterococcus faecium*	Ø	Ø	4 (3.4%)	9 (20.9%)	10 (8.7%)	2 (5.6%)

**Table 2:** Inflammatory markers according to type of pathogen in patients with acquired bacterial LRTI.

	None	Gram+	Gram−	Combined	MDR	Fungal	*p*
WBC (×10^9^/L)							0.496 ^1^
Mean (SD)	12.3 (6.8)	12.2 (3.0)	15.9 (7.2)	7.8 (3.1)	12.4 (5.7)	13.2 (6.4)	
Range	1.9–56.6	7.8–16.0	7.0–26.4	5.4–11.3	2.3–39.2	7.8–23.1	
Neutrophil (%)							0.495 ^1^
Mean (SD)	84.6 (14.9)	91.6 (4.1)	89.9 (6.2)	86.0 (8.4)	87.0 (12.3)	88.4 (8.2)	
Range	6.5–97.8	85.4–95.4	78.8–95.7	76.5–92.4	8.9–96.6	78.8–97.2	
Lymphocyte (%)							0.910 ^1^
Mean (SD)	7.5 (7.4)	4.5 (3.6)	5.8 (4.8)	8.4 (3.9)	6.8 (9.1)	7.2 (7.4)	
Range	0.4–69.4	2.2–10.8	1.5–13.8	5.7–12.9	0.2–87.2	1.3–15.5	
NLR							0.432 ^1^
Mean (SD)	20.8 (24.2)	29.0 (14.1)	29.4 (21.6)	11.9 (5.2)	24.0 (21.6)	35.0 (30.1)	
Range	0.4–235.7	7.9–43.4	5.7–62.8	5.9–15.6	0.2–173.3	5.1–74.8	
CRP (mg/L)							0.426 ^1^
Mean (SD)	129.2 (88.5)	119.9 (65.3)	120.0 (49.2)	107.2 (53.7)	148.8 (94.5)	122.9 (47.4)	
Range	3.0–444.0	48.5–224.3	34.0–181.0	46.9–150.0	3.2–546.1	58.3–163.5	
PCT (ng/mL)							0.950 ^1^
Mean (SD)	4.0 (14.3)	4.9 (10.2)	8.1 (13.8)	1.4 (1.7)	3.6 (11.6)	3.6 (5.2)	
Range	0.0–100.0	0.2–23.1	0.2–42.5	0.1–3.4	0.0–100.0	0.1–12.3	
IL-6 (pg/mL)							0.006 ^1^
Mean (SD)	220.6 (392.3)	199.1 (259.9)	63.4 (103.3)	1011.0 (847.0) *	189.0 (340.0)	113.2 (141.1)	
Range	0.0–1500.0	20.1–648.7	12.0–337.7	33.0–1500.0	0.1–1500.0	0.0–359.3	
Ferritin (mg/L)							0.640 ^1^
Mean (SD)	1395.8 (1202.4)	629.6 (547.4)	1264.7 (913.1)	817.7 (352.9)	1370.1 (1033.0)	1656.0 (1864.5)	
Range	0.0–4500.0	63.0–1305.0	33.0–2696.0	499.0–1197.0	72.0–4500.0	0.0–3746.0	

^1^ Kruskal–Wallis test. * Statistically significant difference after DSCF pairwise comparison.

**Table 3:** Odds ratios of effect of biomarkers of inflammatory response on survival rates in patients with LRT isolates.

LRT		Survivors	Non-Survivors	OR (Univariable)	OR (Multivariable)
Age		65.0 (11.9)	71.3 (10.5)	1.05 (1.02–1.08, *p* = 0.001)	1.06 (1.01–1.12, *p* = 0.025)
Sex	M	32 (14.8)	184 (85.2)	Ref	Ref
	F	15 (17.0)	73 (83.0)	0.85 (0.44–1.69, *p* = 0.626)	1.19 (0.39–3.94, *p* = 0.771)
CCI		4.3 (3.4)	4.7 (2.2)	1.09 (0.94–1.27, *p* = 0.265)	0.96 (0.76–1.24, *p* = 0.726)
WBC	(×10^9^/L)	12.8 (5.8)	12.5 (6.0)	0.99 (0.94–1.05, *p* = 0.773)	1.04 (0.92–1.18, *p* = 0.551)
Neutrophil	(%)	83.2 (16.7)	88.2 (8.8)	1.03 (1.01–1.06, *p* = 0.009)	1.10 (1.03–1.29, *p* = 0.039)
Lymphocyte	(%)	7.0 (5.1)	6.9 (9.7)	1.00 (0.97–1.04, *p* = 0.939)	1.09 (0.98–1.33, *p* = 0.297)
CRP	(mg/L)/10	1.3 (1.0)	1.4 (0.9)	1.24 (0.86–1.87, *p* = 0.263)	0.92 (0.54–1.73, *p* = 0.785)
Procalcitonin	(ng/mL) × 10	21.5 (66.3)	37.7 (107.9)	1.00 (1.00–1.01, *p* = 0.346)	1.00 (1.00–1.01, *p* = 0.916)
Ferritin	(mg/L)/100	8.5 (6.6)	13.8 (10.3)	1.08 (1.03–1.16, *p* = 0.005)	1.13 (1.03–1.25, *p* = 0.016)
IL6	(pg/mL)/100	1.8 (3.8)	2.1 (3.7)	1.03 (0.93–1.19, *p* = 0.644)	1.08 (0.93–1.37, *p* = 0.400)
D-Dimer	(mg/L)	2.8 (1.6)	2.9 (1.4)	1.04 (0.82–1.31, *p* = 0.731)	0.95 (0.64–1.40, *p* = 0.803)

Mean (SD); Model fit: Nagelkerke R^2^ = 0.242, ROC-AUC = 0.801, H&L = Chi^2^ 12.52 (*p* = 0.130).

**Table 4:** Isolated LRT pathogens and mortality rates.

		ICU Mortality		
LRT		Y	N	Total
None	Observed	246	141	387
	% within row	63.6%	36.4%	100.0%
Gram+	Observed	7	3	10
	% within row	70.0%	30.0%	100.0%
Gram−	Observed	10	8	18
	% within row	55.6%	44.4%	100.0%
Combined	Observed	3	0	3
	% within row	100.0%	0.0%	100.0%
MDR	Observed	231	36	267
	% within row	86.5%	13.5%	100.0%
Fungal	Observed	6	1	7
	% within row	85.7%	14.3%	100.0%
Total	Observed	503	189	692
	% within row	72.7%	27.3%	100.0%

χ^2^ 43.36, *p* < 0.001.

**Table 5:** Inflammatory markers according to type of pathogen in patients with positive blood cultures.

	None	Gram+	Gram−	Combined	MDR	*p*
WBC (×10^9^/L)						0.304 ^1^
Mean (SD)	12.2 (6.2)	14.9 (7.5)	18.1 (8.2)	16.4 (3.0)	12.7 (6.6)	
Range	1.9–56.6	6.4–27.6	12.3–23.9	14.3–18.5	4.9–39.2	
Neutrophil (%)						0.980 ^1^
Mean (SD)	86.2 (12.9)	85.5 (17.6)	89.8 (3.6)	88.2 (7.7)	85.3 (15.5)	
Range	6.5–97.6	29.9–95.4	87.3–92.4	82.8–93.7	10.6–97.8	
Lymphocyte (%)						0.619 ^1^
Mean (SD)	6.9 (6.3)	10.1 (18.6)	4.0 (0.8)	7.2 (6.7)	7.8 (12.0)	
Range	0.2–69.4	1.5–69.1	3.5–4.6	2.5–12.0	0.5–87.2	
NLR						0.966 ^1^
Mean (SD)	22.5 (23.9)	27.4 (18.3)	22.7 (5.2)	22.2 (21.6)	22.8 (19.4)	
Range	0.4–235.7	0.4–62.8	19.0–26.4	6.9–37.5	0.2–87.8	
CRP (mg/L)						0.652 ^1^
Mean (SD)	136.3 (91.4)	115.7 (60.5)	193.2 (140.6)	107.0 (7.1)	147.6 (88.2)	
Range	3.0–546.1	25.0–224.3	93.8–292.6	101.9–112.0	24.2–455.2	
PCT (ng/mL)						0.020 ^1^
Mean (SD)	3.0 (9.3)	3.4 (7.1)	19.7 (27.6) *	1.0 (1.0)	8.6 (24.2) *	
Range	0.0–100.0	0.1–23.1	0.2–39.2	0.3–1.7	0.1–100.0	
IL-6 (pg/mL)						0.715 ^1^
Mean (SD)	219.3 (388.2)	133.5 (181.6)	342.0 (405.9)	80.0 (15.1)	160.4 (320.8)	
Range	0.0–1500.0	6.1–648.7	55.0–629.1	69.3–90.7	7.6–1500.0	
Ferritin (mg/L)						0.306 ^1^
Mean (SD)	1365.7 (1157.4)	1361.1 (1083.2)	3090.5 (1993.3)	1331.5 (468.8)	1319.7 (870.9)	
Range	0.0–4500.0	146.0–3746.0	1681.0–4500.0	1000.0–1663.0	72.0–4207.0	

^1^ Kruskal–Wallis test. * Statistically significant difference after DSCF pairwise comparison.

**Table 6:** Pathogens in blood cultures and mortality rates.

		ICU Mortality		
Blood Culture		Y	N	Total
None	Observed	395	164	559
	% within row	70.7%	29.3%	100.0%
Gram+	Observed	17	9	26
	% within row	65.4%	34.6%	100.0%
Gram−	Observed	2	0	2
	% within row	100.0%	0.0%	100.0%
Combined	Observed	3	1	4
	% within row	75.0%	25.0%	100.0%
MDR	Observed	85	15	100
	% within row	85.0%	15.0%	100.0%
Fungal	Observed	1	0	1
	% within row	100.0%	0.0%	100.0%
Total	Observed	503	189	692
	% within row	72.7%	27.3%	100.0%

χ^2^ 10.62, *p* = 0.059.

**Table 7:** Odds ratios of effect of biomarkers of inflammatory response on survival rates in patients with BSI infections.

BSI		Survivors	Non-Survivors	OR (Univariable)	OR (Multivariable)
Age		64.0 (11.4)	70.0 (11.1)	1.05 (1.01–1.09, *p* = 0.022)	0.93 (0.81–1.04, *p* = 0.235)
Sex	M	18 (19.6)	74 (80.4)	Ref	Ref
	F	7 (17.1)	34 (82.9)	1.18 (0.47–3.28, *p* = 0.734)	0.73 (0.07–9.36, *p* = 0.799)
CCI		3.1 (2.4)	4.9 (2.4)	1.44 (1.14–1.90, *p* = 0.005)	2.97 (1.18–11.08, *p* = 0.050)
WBC	(×10^9^/L)	13.9 (6.2)	12.9 (7.1)	0.98 (0.92–1.05, *p* = 0.536)	1.28 (0.97–1.82, *p* = 0.116)
Neutrophil	(%)	83.9 (17.4)	87.1 (11.8)	1.02 (0.98–1.05, *p* = 0.296)	1.01 (0.87–1.27, *p* = 0.921)
Lymphocyte	(%)	6.4 (5.3)	7.8 (12.2)	1.01 (0.97–1.09, *p* = 0.608)	0.98 (0.84–1.27, *p* = 0.828)
CRP	(mg/L)/10	1.2 (0.8)	1.4 (0.8)	1.26 (0.73–2.36, *p* = 0.432)	3.67 (0.79–27.85, *p* = 0.141)
Procalcitonin	(ng/mL) × 10	98.4 (279.4)	62.8 (185.8)	1.00 (1.00–1.00, *p* = 0.451)	0.99 (0.98–1.00, *p* = 0.118)
Ferritin	(mg/L)/100	13.8 (10.0)	14.3 (10.3)	1.00 (0.96–1.06, *p* = 0.850)	0.96 (0.79–1.16, *p* = 0.639)
IL6	(pg/mL)/100	2.1 (4.5)	1.6 (2.6)	0.95 (0.82–1.12, *p* = 0.501)	1.56 (0.77–5.74, *p* = 0.341)
D-Dimer	(mg/L)	3.0 (1.5)	2.9 (1.4)	0.95 (0.68–1.31, *p* = 0.749)	0.62 (0.28–1.24, *p* = 0.199)

Mean (SD). Model fit: Nagelkerke R^2^ = 0.469, ROC-AUC = 0.865, H&L = Chi^2^ 3.62 (*p* = 0.89).

**Table 8:** Inflammatory markers according to type of pathogen in patients with positive urine cultures.

	None	Gram+	Gram−	Combined	MDR	Fungal	*p*
WBC (×10^9^/L)							0.984 ^1^
Mean (SD)	12.5 (6.6)	12.3 (5.2)	11.7 (5.0)	8.8 (N/A)	12.0 (5.4)	12.6 (4.1)	
Range	1.9–56.6	6.5–21.4	6.6–26.4	8.8–8.8	5.1–26.4	9.2–22.8	
Neutrophil (%)							0.565 ^1^
Mean (SD)	85.4 (14.5)	88.9 (4.6)	88.8 (5.1)	78.7 (N/A)	89.2 (7.1)	88.8 (5.0)	
Range	6.5–97.6	82.1–94.7	79.5–94.2	78.7–78.7	64.0–97.8	81.6–96.4	
Lymphocyte (%)							0.937 ^1^
Mean (SD)	7.3 (8.7)	6.9 (4.2)	6.6 (4.5)	10.7 (N/A)	5.9 (5.3)	5.8 (3.1)	
Range	0.2–87.2	2.5–14.3	1.5–15.3	10.7–10.7	1.2–20.6	1.5–11.9	
NLR							0.928 ^1^
Mean (SD)	22.6 (24.0)	18.4 (11.9)	22.7 (18.1)	7.4 (N/A)	26.2 (17.9)	23.0 (18.5)	
Range	0.2–235.7	5.7–37.5	5.3–62.8	7.4–7.4	3.1–68.5	6.9–64.3	
CRP (mg/L)							0.531 ^1^
Mean (SD)	135.7 (90.5)	134.1 (83.2)	110.7 (68.7)	111.0 (N/A)	160.1 (75.0)	164.3 (130.8)	
Range	3.0–546.1	25.0–244.2	4.7–266.8	111.0–111.0	24.1–314.0	44.9–455.2	
PCT (ng/mL)							0.325 ^1^
Mean (SD)	3.9 (12.9)	2.6 (3.2)	1.6 (3.7)	0.1 (N/A)	2.2 (3.6)	12.3 (29.4)	
Range	0.0–100.0	0.1–8.7	0.1–14.3	0.1–0.1	0.1–15.7	0.1–100.0	
IL-6 (pg/mL)							0.880 ^1^
Mean (SD)	204.6 (363.6)	171.7 (114.3)	162.7 (363.8)	14.6 (N/A)	285.1 (520.7)	185.4 (340.9)	
Range	0.0–1500.0	68.0–331.8	7.3–1391.3	14.6–14.6	7.4–1500.0	13.5–1202.0	
Ferritin (mg/L)							0.602 ^1^
Mean (SD)	1396.1 (1131.4)	1238.0 (1033.4)	877.4 (1183.2)	1192.0 (N/A)	1471.0 (1184.6)	1149.2 (517.0)	
Range	0.0–4500.0	209.0–3289.0	9.0–4500.0	1192.0–1192.0	65.0–4500.0	206.0–1819.0	

^1^ Kruskal–Wallis test.

**Table 9:** Pathogens in urine cultures and ICU mortality rates.

		ICU Mortality		
Urine Culture		N	Y	Total
None	Observed	163	409	572
	% within row	28.5%	71.5%	100.0%
Gram+	Observed	5	16	21
	% within row	23.8%	76.2%	100.0%
Gram-	Observed	9	13	22
	% within row	40.9%	59.1%	100.0%
Combined	Observed	1	3	4
	% within row	25.0%	75.0%	100.0 %
MDR	Observed	9	42	51
	% within row	17.6%	82.4%	100.0%
Fungal	Observed	2	20	22
	% within row	9.1%	90.9%	100.0%
Total	Observed	189	503	692
	% within row	27.3%	72.7%	100.0%

χ^2^ 8.6722, *p* = 0.123.

## Data Availability

All data generated or analyzed during this study are included in this published article.
